# Plant Genomics in the 21^st^ Century

**DOI:** 10.2174/138920210790217963

**Published:** 2010-03

**Authors:** Chhandak Basu

It is my great pleasure as a Guest Editor of the journal Current Genomics to present you with a ‘hot topic issue’ on plant genomics. The complete sequence of genome of model plant *Arabidopsis thaliana* was a boost for plant genomics research. Following the path of *Arabidopsis* sequencing, hundreds of plant genomes are being sequenced and we have ocean of sequence data. It is a challenge for us to interpret the functions of thousands of genes. Surely, computational biology will be a great help in predicting functions of plant genes. Publicly available web based sequence annotation softwares are another resource to annotate and characterize plant genes. It is a challenge and responsibility for plant biologists to provide food, fiber and fuel for this exponentially increasing population. Sequences of model plants and crop plants will help us to understand the genome architectures of the plants and will help us to identify economically important genes. It is a well-established fact that a great alternative for whole genome sequencing is development of an EST (Expressed Sequence Tags) database. The ESTs will give us a snapshot of whole genome with a small percentage of cost of whole genome sequencing. The next generation sequencing including 454 sequencing (Roche Applied Sciences), Solexa system (Illumina) and SOliD system (Applied Biosystems) are also great resources for plant genomicists. These technologies will generate enormous amount of sequencing data, which will be beneficial to understand the basic physiology of plants.

This issue of Current Genomics is a compilation of review articles on various facets of plant genomics written by experts. Das and Pandey discussed roles of calcium sensing proteins such calmodulin in abiotic stress response in plants. They especially discussed the roles of calcium dependent kinase genes in rice. Lelandais-Brière *et al*. elaborated the roles of micro RNAs, a group of non coding RNAs, in root development. Micro RNAs and short interfering RNAs are very powerful tools to suppress gene expressions and surely will become more effective means to study plant genomes since we have more and more plant genome sequence information available. Volk described how some of the tools of genomics could be used to study plant cryobiology. Microarray technology could be used to study gene expression in cryostressed plants. This will help us to understand plant stress response in cryogenic conditions and will help us to design better plant germplasm conservation strategies. Panthee and Cheng discussed use of molecular markers, and tools of transcriptomics, proteomics and metabolomics to study fungal disease in tomatoes. They highlighted how tools of genomics and other transgenic approaches could be implemented to develop disease resistant tomato cultivars. Zhang *et al.* presented a systems biology approach to study protein-protein interactome in plants. They explained how *in silico* methods along with experimental approaches like yeast two hybrid systems, affinity purification and mass spectrometry, biomolecular fluorescence complementation etc. could be used to understand the proteome of plants. Horvath discussed on applications of tools of genomics to understand the weediness traits of weeds. In particular, the author focused on use of EST databases and microarrays to understand the biology of weeds. Understanding the genomic architecture of weeds will help us to develop better weed control strategies.

I would like to sincerely thank all the reviewers from across the globe for their valuable suggestions to improve the quality of research articles. Special thanks to Editor-in-Chief Dr. Christian Néri for encouragement for this special issue. It was a great opportunity for me to interact with scientists from all over the world including USA, India and France. I am convinced this issue will be useful for the plant scientists, academicians, industry professionals, students and plant genomics enthusiasts. I will be looking forward to editing another issue on plant genomics in future.

